# Intracardiac thrombus in a child with behçet’s disease

**DOI:** 10.1186/1546-0096-12-S1-P365

**Published:** 2014-09-17

**Authors:** Selçuk Yüksel, Tülay Becerir, Havva Evrengül, Ali Koçyiğit, Yasemin Işık Balcı, Aziz Polat, Dolunay Gürses, Mustafa Doğan

**Affiliations:** 1 Pediatric Rheumatology, Pamukkale University School of Medicine, DENIZLI, Turkey; 2 Radiodiagnostic, Pamukkale University School of Medicine, DENIZLI, Turkey; 3 Pediatric Hematology, Pamukkale University School of Medicine, DENIZLI, Turkey; 4 Pediatric Cardiology, Pamukkale University School of Medicine, DENIZLI, Turkey

## Introduction

Behçet’s disease (BD) is recognized as a systemic vasculitis involving both arteries and veins of any size and characterized by recurrent oral aphthous ulcers, genital ulcers, and uveitis and skin lesions. The mean age of onset is in the fourth decade.

## Objectives

Childhood BD accounts for 1–3% of all cases. Intracardiac thrombus in BD is rarely seen. We present an adolescent with a severe manifestation BD who had an intracardiac thrombus.

## Methods

A sixteen-year old male patient was admitted with painful localized swelling of the legs, high fever and cough. Medical history revealed that he had suffered from recurrent oral ulcers for 5 years, recurrent painful nodules on his legs and uveitis in the last one year. He was diagnosed with BD at another center; colchicine and eye drops were started. However, he didn’t use the pills and eye drops, regularly. Family history revealed a maternal uncle who died due to BD.

## Results

His height and weight were below 3%. Oral ulcers, vision loss in the left eye and erythema nodosum on the lower extremities were observed. Eye examination showed a serious sequel of uveitis, but there was no evidence of active uveitis. Although azathioprine, prednisolone and colchicine were prescribed, he did not use the medications, regularly. After 3 months, he applied with chest pain, cough and abdominal pain. Physical examination showed pale appearance, slightly hyperemic tonsils, postnasal drip. Laboratory investigations showed; Hgb: 12.7 g/dl, Plt:487000 K/uL, WBC:11700 K/uL, AST:71 IU/L, ALT:141 IU/L, C-RP:1,7 mg/dL, and erythrocyte sedimentation rate: 40 mm/hr. Abdominal ultrasound showed many lymph nodes (largest one 10 x 15 mm), and chest X-ray demonstrated irregularity and notches in the right diaphragm. Transthoracic echocardiography showed a 15 x 8 mm mass linked to trabecular muscle in the right ventricle. Thorax CT angiography showed a hypodense filled defect of 22 x 9 mm in the right ventricle with fibrosis-focal consolidation areas in the bilateral lower lobes of the lungs, and micro nodules with frosted glass opacity in the superior segment of the lower right lobe. With the diagnosis of pulmonary micro emboli and intracardiac thrombus, low molecular weight heparin, pulse methylprednisolone (3 consecutive days), oral prednisolone 2 mg/kg/day and pulse cyclophosphamide (for 6 doses-monthly) were started. Clinical improvement was observed and control echocardiography showed reduction in the size of the mass in the right ventricle. Steroid (with reducing doses), colchicine and anticoagulant treatment were continued. During this period oral ulcers and erythema nodosum did not recur and there were no active uveitis attacks. Thrombosis panel showed heterozygote mutations of an allele of factor V Leiden (G1691A) and prothrombin (G20210A). A control thorax CT angiography in the 5th month of treatment showed that intracardiac mass had disappeared, regression of the fibrosis-focal consolidation areas in the parenchyma of both lungs and the frost-glass opacity nodule had disappeared.

## Conclusion

In Behcet’s disease intracardiac thrombus are rarely seen. However it is related with high mortality and morbidity. Thus, early diagnosis and treatment is very important from the point of view of prognosis. Instead of surgical treatment intense immunosuppressive treatment with anticoagulant therapy may be more beneficial in these type of patients.

## Disclosure of interest

None declared

